# Stem cells of the suture mesenchyme in craniofacial bone development, repair and regeneration

**DOI:** 10.1038/ncomms10526

**Published:** 2016-02-01

**Authors:** Takamitsu Maruyama, Jaeim Jeong, Tzong-Jen Sheu, Wei Hsu

**Affiliations:** 1 Department of Biomedical Genetics, Center for Oral Biology, University of Rochester Medical Center, Rochester, 14642 New York, USA; 2 Stem Cell and Regenerative Medicine Institute, University of Rochester Medical Center, Rochester, 14642 New York, USA; 3 Center for Musculoskeletal Research, University of Rochester Medical Center, Rochester, 14642 New York, USA; 4 Wilmot Cancer Institute, University of Rochester Medical Center, Rochester, 14642 New York, USA

## Abstract

The suture mesenchyme serves as a growth centre for calvarial morphogenesis and has been postulated to act as the niche for skeletal stem cells. Aberrant gene regulation causes suture dysmorphogenesis resulting in craniosynostosis, one of the most common craniofacial deformities. Owing to various limitations, especially the lack of suture stem cell isolation, reconstruction of large craniofacial bone defects remains highly challenging. Here we provide the first evidence for an Axin2-expressing stem cell population with long-term self-renewing, clonal expanding and differentiating abilities during calvarial development and homeostastic maintenance. These cells, which reside in the suture midline, contribute directly to injury repair and skeletal regeneration in a cell autonomous fashion. Our findings demonstrate their true identity as skeletal stem cells with innate capacities to replace the damaged skeleton in cell-based therapy, and permit further elucidation of the stem cell-mediated craniofacial skeletogenesis, leading to revealing the complex nature of congenital disease and regenerative medicine.

## Abstract (web summary)

The suture mesenchyme has been postulated to act as the niche for stem cells for calvarial bones but the identity of the stem cells is unknown. Here, Maruyama *et al.* suggest that Axin2 expressing cells act as stem cells not only in craniofacial bone development and homeostasis but in injury-induced repair.

## Graphical abstract

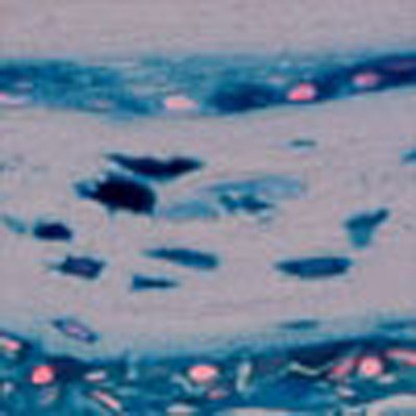

Craniofacial bones are formed mainly through intramembranous ossification, a mechanism different from endochondral ossification required for development of the body skeleton^1^. The skeletal structures are quite distinct between the two, thus they are likely to have their own unique stem cell populations^2^. The calvarial sutures serve as the growth centre critical for healthy development of the craniofacial skeleton^3^. Defects in suture morphogenesis cause its premature closure, resulting in development of craniosynostosis, a disease frequently associated with facial deformity, mental retardation and problems in vision, hearing and breathing^4^. It has long been postulated that the suture mesenchyme is the niche of skeletal stem cells essential for calvarial morphogenesis^5^,^6^,^7^. However, very limited knowledge is available for suture biology and suture stem cells (SuSCs) have yet to be isolated.

The bone marrow has long been shown to contain the osteogenic cell population for the body skeleton^8^,^9^. Recent studies have begun to uncover the nature of skeletogenic/skeletal stem cells qualified for the more rigorous stem cell definition^10^,^11^,^12^,^13^,^14^. In the calvarium, there is every expectation that the suture is the niche for stem cells which regulate calvarial bone development. This is further supported by a recent report of Gli1-expressing cells contributing to calvarial maintenance and injury repair using cell tracing analysis^6^. However, stem cells of the calvarial bones have yet to be isolated, and their innate ability to regenerate bones is still unknown^6^. The identity and characteristics of SuSCs, responsible for calvarial bone formation and capable of regenerating craniofacial skeletons, are highly limited to date.

Large craniofacial bone defects caused by various conditions, including cancer surgeries, congenital malformation, trauma and progressive deforming diseases, are major health issues^15^. The only solution for such extensive skeletal injuries is to undergo a reconstructive operation. Current strategies use autologous grafts, allogenic grafts or alloplastic materials to enhance bone regeneration and to restore craniofacial elements^16^. However, success of these reconstructions remains highly challenging owing to a number of limitations. This has led to exploration of alternative approaches, especially stem cell-based therapy^17^,^18^. Cellular components, either transplanted from an exogenous source or recruited from local stem/progenitor cells, must be present at the recipient site to give rise to the new structural tissues. However, the lack of knowledge in SuSCs greatly restricts further advances. Their isolation is likely to benefit craniofacial reconstruction and to advance the field of regenerative medicine.

In this study, we identify, isolate and characterize a SuSC population that expresses high levels of Axin2 and qualifies as stem cells under a rigorous definition. These naive cells exhibit long-term self-renewing, clonal expanding and differentiating abilities, and behave like *bona fide* stem cells—not only in craniofacial bone development and homoeostasis, but also in skeletal repair and cell-based regenerative therapy.

## Results

### Identification of slow-cycling cells in suture mesenchyme

**Figure 1:**
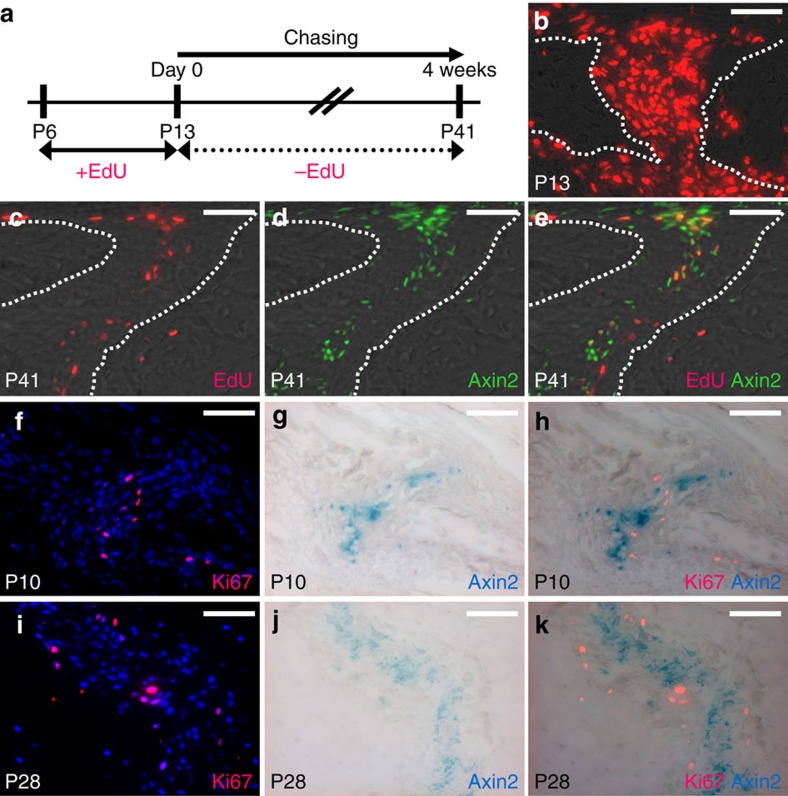
Slow cycling cells expressing Axin2 reside in the midline of the suture mesenchyme. (**a**) An illustration describes pulse-chase labelling analysis for identification of label-retaining cells. Sections of the pulse-chased *Axin2*^*GFP*^ skull were analyzed by EdU staining before (**b**) and after the 4-week chase period (**c**), at P13 and P41, respectively. (**d**) The Axin2-expressing cells were detected by GFP. (**e**) Merged imaging examines cells positive for EdU and Axin2 expression. Sections of the skull were analyzed for cells active in mitotic division and expressing Axin2 by immunostaining of Ki67 and β-gal staining of lacZ, respectively, at P10 (**f**–**h**) and P28 (**i**–**k**). Images are representations from three independent experiments. Scale bar, 50 μm (**b**–**k**).

Quiescence of stem cells is important to ensure lifelong tissue maintenance and to prevent them from premature exhaustion^19^. Taking advantage of their quiescent nature in cell division, hair follicle^20^,^21^ and colon^22^ stem cells were identified by their ability to retain the signal used for DNA incorporation analysis. Therefore, we examined the possible existence of label retaining cells (LRCs) during calvarial development. After pulse labelling for 1 week, most of the mesenchymal cells were marked by EdU (5-ethynyl-2'-deoxyuridine) in the sagittal suture (Fig. 1a,b). Upon chasing for 4 weeks, a small number of LRCs could be detected in the midline of the skeletogenic mesenchyme, a potential niche for skeletal stem cells (Fig. 1a,c). Our prior study of Axin2 revealed its expression in this location during calvarial morphogenesis^5^. In addition, the Wnt downstream genes have been successfully used to identify tissue-specific stem cells^23^,^24^,^25^,^26^. As a direct transcriptional target of β-catenin, Axin2 is also essential to orchestrate the signalling interplay of Wnt, BMP and FGF in mesenchymal cell fate determination^5^,^27^. Therefore, we investigated if Axin2 is expressed in these LRCs. Using the *Axin2*^*GFP*^ model (Supplementary Fig. 1a)^5^,^28^, we found that Axin2 is expressed in all calvarial sutures except the posterior frontal suture, the only one fused at this stage (Supplementary Fig. 2a–d). This differential expression is consistent with our previous finding that Axin2-expressing cells are present in the patent posterior frontal suture, but gradually diminish upon its closure^5^. The link of suture patency to the presence of Axin2 thus implies its crucial role in suture morphogenesis and calvarial development. More importantly, the expression of Axin2 is restricted to the midline of the suture mesenchyme (Fig. 1d, Supplementary Fig. 2), co-localized with cells retaining EdU (Fig. 1e). This highly specific pattern of expression, first detected at postnatal day 9 (Supplementary Fig. 1a), was maintained in the adults, suggesting potential marking of the stem cells residing in the skeletogenic niches.

To test whether the Axin2-expressing cells are active proliferating or slow cycling in nature, we labelled cells undergoing mitotic division with Ki67 and expressing Axin2 using the *Axin2*^*Cre-Dox*^*; R26RlacZ* model (Fig. 1f–k, Supplementary Fig. 1b). The majority of the Ki67+ cells surrounding the Axin2-expressing cells but did not express Axin2 at P10 (Fig. 1f–h; 91±2%, *n*=3, mean±s.e.m.) and P28 (Fig. 1i–k; 90±2.5%, *n*=3, mean±s.e.m.), suggesting the use of Axin2 as a marker to identify slow cycling stem cells present in the midline of the suture mesenchyme, a niche with no expression of known osteogenic and chondrogenic markers.

### Self-renewal and differentiation of Axin2-expressing cells

**Figure 2:**
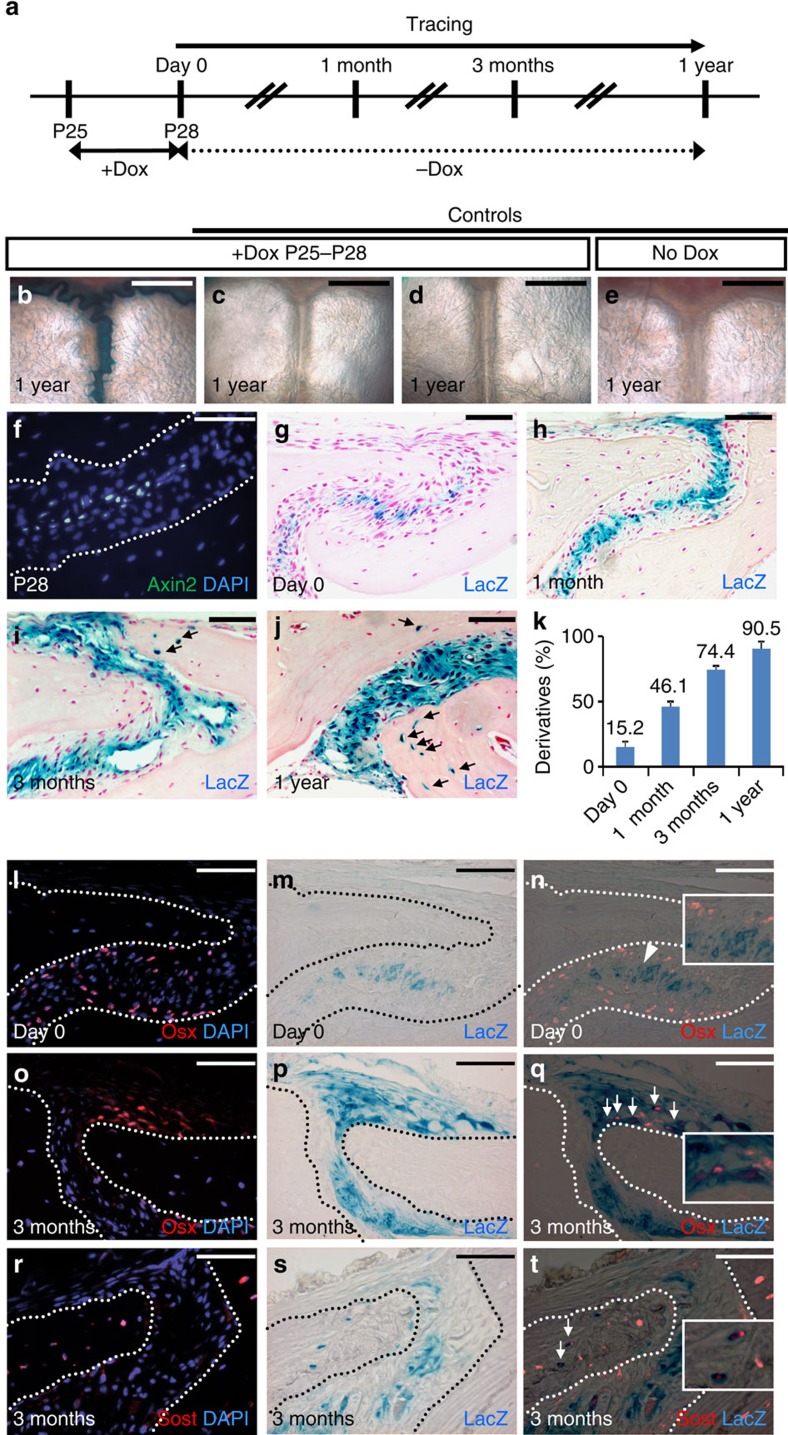
Fate mapping analysis reveals a population exhibiting stem cell characteristics in skeletal homeostastic maintenance. (**a**) A diagram illustrates spatiotemporal-specific tracing of the Axin2-expressing cells. β-gal staining of the skulls examines the Axin2-expressing cells and their descendants before tracing (Day 0; **g**,**m**), or after tracing for 1 month (**h**), 3 months (**i**,**p**,**s**) and 1 year (**b**–**e**,**j**). The Axin2-expressing cells are detected by GFP analysis at P28 before the start of tracing (**f**). Immunostaining analysis of Osterix (Osx) and Sost identifies osteogenic cell types before tracing (**l**) and after 3 months tracing (**o**,**r**). Double labelling analysis examines the expression of osteogenic markers in the Axin2-expressing cells and their derivatives before tracing (**n**) and after 3 months tracing (**q**,**t**). Statistical analysis indicates the ratio of the lacZ-positive cells during the 1-year-tracing period (**k**, ∼250 cells counted, *n*=3, mean±s.e.m.). Arrows and arrowhead indicate lacZ-expressing cells positive and negative for osteogenic marker, respectively. Genotypes: *Axin2rtTA; TRE-Cre; R26R* (**b**,**e**,**g**–**j**,**l**–**t**), *Axin2-rtTA; R26R* (**c**), *TRE-Cre; R26R* (**d**) and *Axin2-rtTA; TRE-H2BGFP* (**f**). Images are representatives of three (**f**,**l**–**t**) and five (**b**–**e**,**g**–**j**) independent experiments. Scale bar, 1 mm (**b**–**e**). Scale bar, 50 μm (**f**–**j**,**l**–**t**).

To determine the self-renewing ability of the Axin2-expressing cells and their contributions to calvarial development and homeostastic maintenance, a series of cell tracing analyses were performed using genetically modified mouse models. We use the *Axin2*^*Cre-Dox*^*; R26RlacZ* model^5^,^29^, which permits spatiotemporal-specific expression of Cre to genetically label the Axin2-expressing cells and examine their fates (Supplementary Fig. 1b). Although Axin2 is a well-known transcriptional target of β-catenin, this model does not measure the Wnt signalling responsiveness. This is because of the withdrawal of doxycycline after a transient labelling. Therefore, the labelled cells do not represent activation of Wnt signalling. First, we performed genetic cell-labelling analysis at P28 (Fig. 2a) because acute development of the skull is completed by this time^30^. Whole-mount β-gal staining revealed a highly regulated expression of the lacZ reporter, suggesting the specificity of our experimental system (Fig. 2b–e). In the *Axin2*^*Cre-Dox*^ mice, the administration of doxycycline for 3 days induced the Cre-mediated expression of the *R26RlacZ* reporter which shows the Axin2-expressing cells in the midline (Fig. 2g), highly resembling the GFP expression pattern (Fig. 2f) before tracing. The number of lacZ positive cells increased in the skeletogenic mesenchyme (Fig. 2h) after a 1-month tracing, which expanded much more widely and were embedded in the bone plate after 3-month and 1-year tracing in the sagittal suture (Fig. 2i,j). Derivatives of the Axin2-expressing cells continued to accumulate over a long tracing period showing no sign of diminishing (Fig. 2k, ∼250 cells counted, *n*=3, mean±s.e.m.). Even after >1-year tracing, the Axin2-expressing cells and their derivatives remain detectable and this population continues to increase in all sutures except for the posterior frontal suture (Supplementary Fig. 3a–f). This is because the posterior frontal suture normally fuses in juveniles and no longer contains the skeletogenic mesenchyme in adults. We noticed significant contributions of the Axin2-expressing cells and their derivatives to all patent sutures and calvarial bones (Fig. 2j,k, Supplementary Fig. 3b–e,i). Their contributions were lower in the central region of frontal and parietal bones further away from the suture mesenchyme (Supplementary Fig. 3g,h). Furthermore, when we examined the body skeleton, very little contribution of this population to homoeostasis of the long bone could be identified (Supplementary Fig. 4a–c). No lacZ-positive signals are detectable in the osteoblasts and osteocytes, suggesting that Axin2 is not able to mark skeletal stem cells in the long bone. In the calvaria, double labelling of the lacZ-positive cells with Osterix and Sost, markers for osteoprogenitors and osteocytes, respectively, indicated that they are precursor cells negative for the Osterix (Fig. 2l–n), but differentiate into osteoprogenitors and osteocytes after 3-month tracing (Fig. 2o–t). Furthermore, the lacZ-positive cells were still maintained in the midline (Fig. 2g–j). Stem cell characteristics of the Axin2-expressing cells were shown in their capabilities to self-renewal and continually give rise to mature cell types over a 1-year monitoring period.

**Figure 3:**
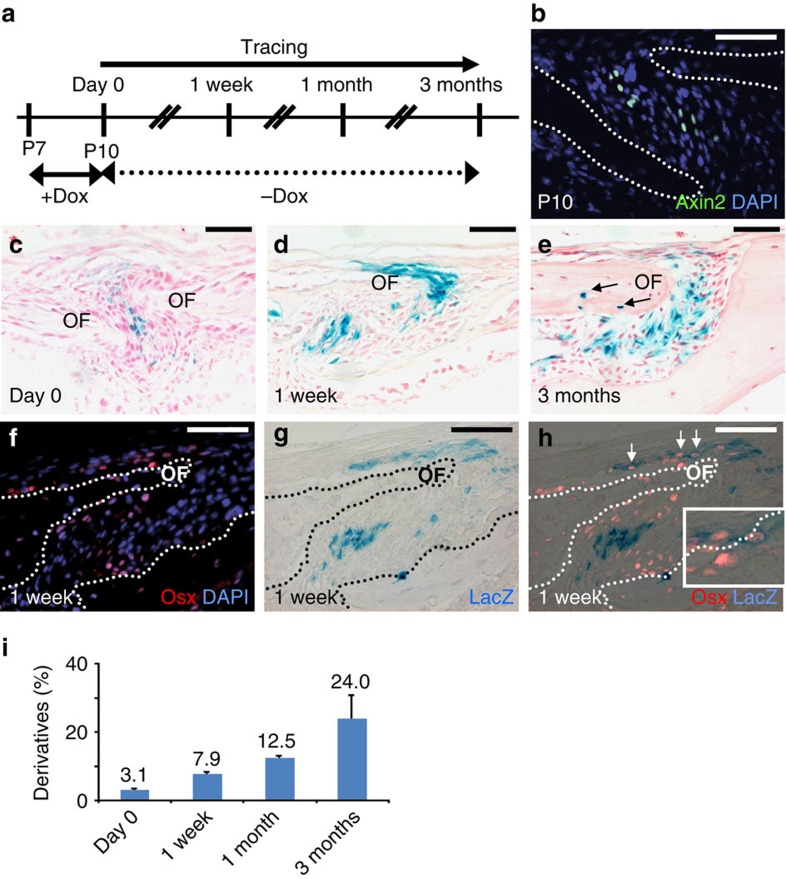
Cells expressing Axin2 are capable of self-renewal and differentiation into osteogenic cell types during skeletal development. (**a**) A diagram illustrates tracing of the Axin2-expressing cells in developing calvarium. GFP analysis detects the Axin2-expressing cells at P10 before the start of tracing (**b**). The Axin2-expressing cells and their descendants before tracing (Day 0; **c**), or after tracing for 1 week (**d**) and 3 months (**e**) are identified by β-gal staining. Double labelling analysis examines the expression of Osx in the Axin2-expressing cells and their descendants after 1 week tracing (**f**–**h**). Statistical analysis indicates that the ratio of the lacZ-positive cells gradually increases over time (**i**, ∼300 cells counted, *n*=3, mean±s.e.m.). Genotypes: *Axin2-rtTA; TRE-H2BGFP* (**b**) and *Axin2rtTA; TRE-Cre; R26R* (**c**–**h**). Images are representatives of three independent experiments. Scale bar, 50 μm (**b**–**h**). OF, osteogenic front.

We next examined the behaviour of the Axin2-expressing cells during early development of the calvarial skeleton which grows rapidly in the first month using the GFP labelling and cell tracing models (Fig. 3a, Supplementary Fig. 1a,b). At P10, GFP and β-gal staining analyses showed the Axin2-expressing cells are restricted to the midline (Fig. 3b,c), highly reminiscent to that observed at P28 (Fig. 2f,g). In 1 week and 3 months after tracing, we were able to detect very similar expansion and differentiation of this cell population into osteogenic cell types (Fig. 3d–h). Overall, the percentage of the Axin2-expressing cells and their descendants increased from 3.1 to 7.9% after 1 week, to 12.5% after 1 month, and to 24% after 3 months (Fig. 3i, ∼300 cells counted, *n*=3, mean±s.e.m.). The results suggest that the Axin2-expressing cells maintain their localization in the niches, and produce skeletogenic descendants over an extensive time period during skeletal development (Fig. 3) and homoeostasis (Fig. 2). Thus, this newly identified cell population potentially acts as developmental and adult SuSCs responsible for calvarial health.

### SuSCs in injury repair

**Figure 4:**
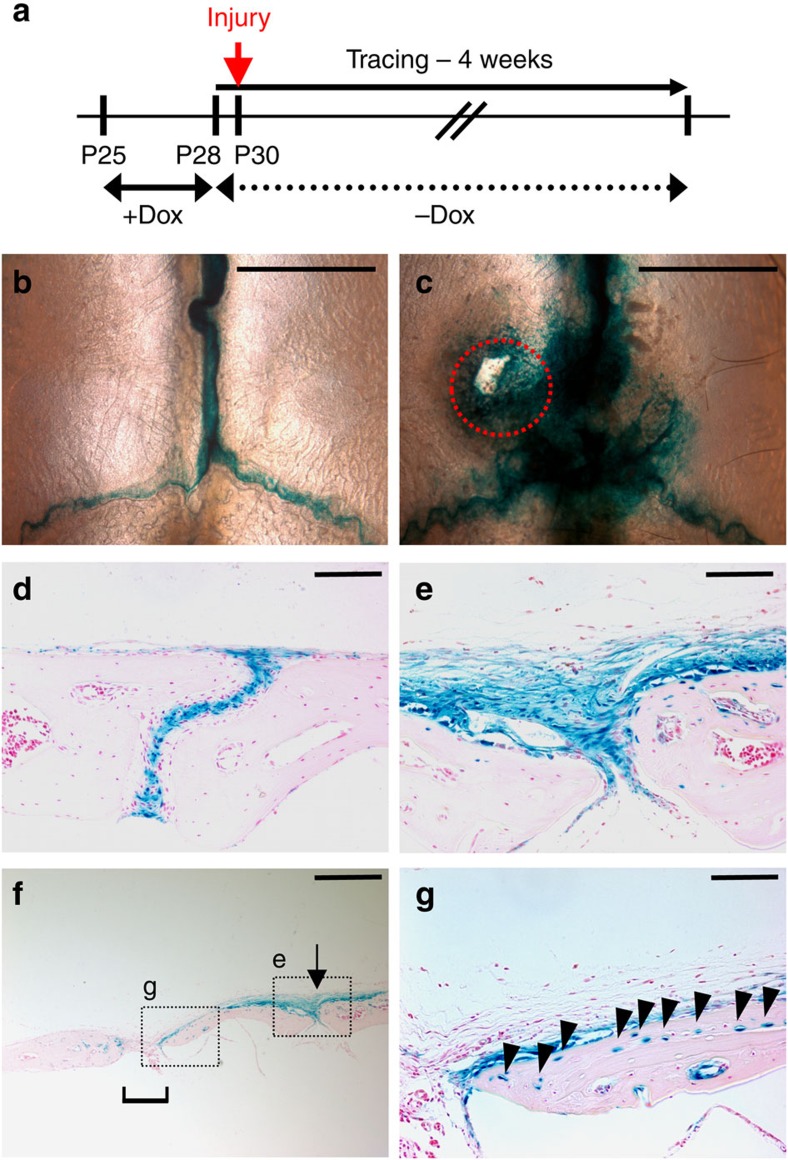
Injury-induced expansion of Axin2-expressing cells undergoing osteoblast differentiation for skeletal repair. (**a**) A diagram illustrates our strategy to map the Axin2-expressing cell fate in an injury repair model. Tracing analysis assesses the population of the Axin2-expressing cells and their descendants with (**c**,**e**–**g**) or without (**b**,**d**) injury in whole mounts (**b**,**c**) and sections (**d**–**g**) of the β-gal-stained *Axin2*^*Cre-Dox*^*; R26R* mice. Bracket, arrow and arrowheads indicate the unhealed region, suture mesenchyme and osteocytes in newly regenerated bones, respectively. Images are representatives of three independent experiments. Scale bar, 2 mm (**b**,**c**). Scale bar, 100 μm (**d**,**e**,**g**). Scale bar, 400 μm (**f**).

Adult stem cells are known to be involved in the healing processes during tissue damage^31^,^32^,^33^. To examine the involvement of the Axin2-expressing cells in skeletal regeneration, we performed the genetic cell-labelling study in an injury repair model. Using the *Axin2*^*Cre-Dox*^*; R26RlacZ* cell tracing model, we created a hole with a 1.4 mm micro-drill on the right parietal bone at P30, 2 days after tracing (Fig. 4a). Four weeks after the operation, we detected a drastic expansion of the lacZ-positive cells surrounding the skeletogenic mesenchyme (Fig. 4b–e). Furthermore, these cells moved into the damaged site, and developed into osteocytes embedded in the bone plate (Fig. 4f,g). Double labelling analysis revealed that the osteoprogenitors and osteocytes positive for Osx and Sost, respectively, originate from those expressing Axin2 (Supplementary Fig. 5). This data suggests that the Axin2-expressing cells expand and their derivatives differentiate into osteogenic cell types, and contribute directly to bone regeneration in response to skeletal injury. Thus further supports the Axin2-expresing cells containing adult SuSCs.

### Regenerating ability of SuSCs

To further our characterization of these potential SuSCs, we performed bone regeneration by transplantation analysis. Cells isolated from the P28 suture mesenchyme were implanted into the kidney capsule to assess their regenerating ability. After transplantation for 6 weeks, staining with hematoxylin and eosin, von Kossa and pentachrome, demonstrated formation of the ectopic bone in the recipients implanted with 10^5^ cells containing SuSCs but not the control (Supplementary Fig. 6a–h). The ectopic bones with limited bone marrow space appear to be intramembranous bones resembling the calvarial bone plates, which differ from the ectopic bones generated by transplantation of the cortical bone cells isolated from the femur and tibia (Supplementary Fig. 6i,j). These results are in agreement with a prior report describing that embryonic cells isolate from the body and calvarial skeletons can generate bones with distinct structures resembling endochondral and intramembranous bones, respectively^34^. However, the absence of bone marrows is not a feature for the regenerated intramembranous bones suggested previously^34^.

**Figure 5:**
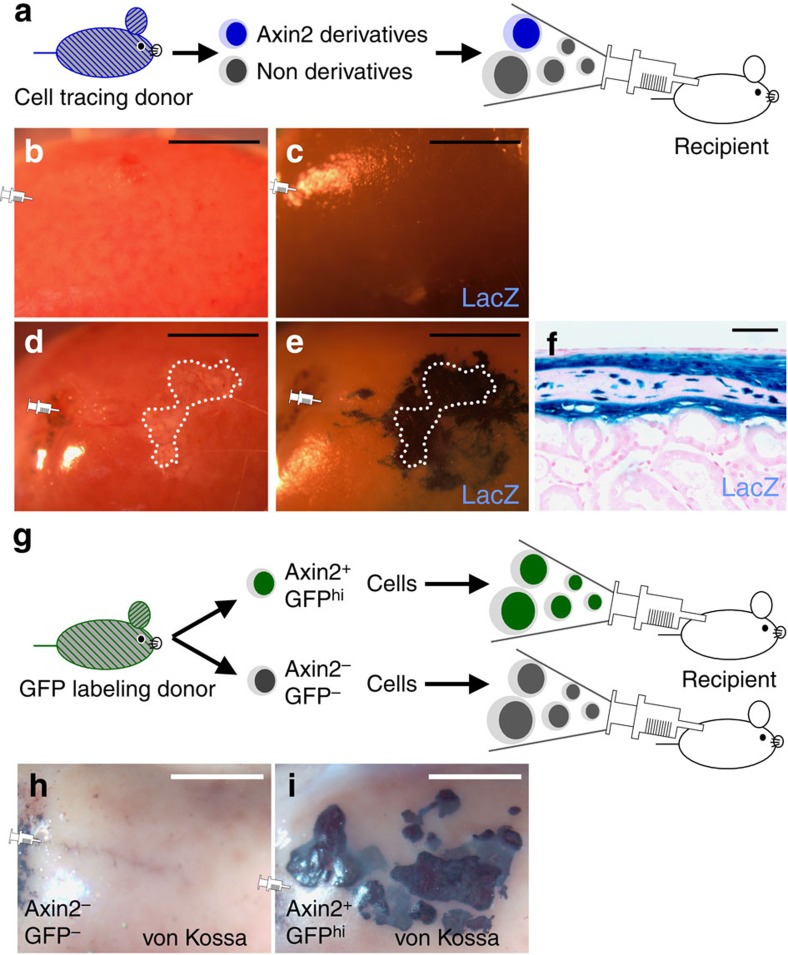
Skeletal stem cells residing in the suture mesenchyme are enriched in the Axin2-expressing cell population. Diagrams illustrate our strategies to examine whether the regenerated bone originates from the Axin2-expressing cells (**a**) and to determine their regenerative potential (**g**) using the cell tracing and GFP labelling models, respectively. Transplantation with inclusion of no cell (**b**,**c**), or suture mesenchymal cells (**d**–**f**) isolated from the *Axin2*^*Cre-Dox*^*; R26RlacZ* mice, examines contribution of the Axin2-expressing cells and their derivatives, stained positive for β-gal, to the ectopic bone formation. Regeneration analysis is performed on the Axin2^−^ (**h**) and Axin2^+^ (**i**) cell populations, isolated from the *Axin2*^*GFP*^ calvaria, after their separation based on the differential expression of GFP. The transplanted kidneys are examined by gross evaluation (**b**,**d**), β-gal staining in whole mount (**c**,**e**) and sections (**f**) and von Kossa staining (**h**,**i**). Images in **b**–**f** and **h**–**i** are representatives of three independent experiments. Scale bar, 2 mm (**b**–**e**,**h**–**i**). Scale bar, 50 μm (**f**).

Next, to examine cellular origin of the regenerated bone, potentially derived from Axin2-expressing cells, a combination of cell tracing and kidney capsule transplantation studies were performed. First, 50,000 cells, isolated from the P28 *Axin2*^*Cre-Dox*^*; R26RlacZ* suture mesenchyme, were transplanted into the kidney capsule without prior separation/purification of the labelled cells (Fig. 5a). However, the use of the cell tracing model (Supplementary Fig. 1b) permitted an easy identification of the Axin2-expressing cells and their derivatives, which are positive for β-gal staining. Two weeks after the transplantation, we found that the majority of the regenerated bones derived from the Axin2-expressing cells (Fig. 5b–f) according to the amount of osteocytes (88±5.4%, *n*=3, mean±s.e.m.). The osteoprogenitors and osteoblasts, as well as osteocytes embedded in the bone plate are positive for β-gal staining (Fig. 5f, Supplementary Fig. 7a–f) suggesting that the Axin2-expressing cells are the predominant source of SuSCs in the suture mesenchyme. In contrast, there is a very limited contribution of Axin2-expressing cells isolated from cortical bones of the femur and tibia to the regenerated bones (Supplementary Fig. 4d). We then furthered our analysis by kidney capsule transplantation of either the Axin2-expressing cell population with high levels of GFP (Axin2^+^/GFP^hi^), or the non-expressing cell population negative for GFP (Axin2^−^/GFP^−^) isolated from the P28 *Axin2*^*GFP*^ suture mesenchyme (Fig. 5g, Supplementary Figs 1a,7g). Transplantation of 100,000 Axin2^+^/GFP^hi^ cells, but not Axin2^−^/GFP^−^ cells, was able to generate ectopic bones (Fig. 5h,i). These results further support inclusion of SuSCs with long-term self-renewing, differentiating and regenerating abilities in the Axin2-expressing cell population.

To test if the Axin2-expressing cell population is similar to skeletal stem cells found in the bone marrow, we examine the expression of Mcam (CD146), Nestin, Leptin receptor (Lepr) and Gremlin1 (refs 10, 12, 13, 14). Comparing gene-expression profile of the Axin2^+^/GFP^hi^ and Axin2^−^/GFP^−^ cell populations by microarray, and then by quantitative real-time RT-PCR, we found that with the exception of Lepr, none of these bone marrow-derived skeletal stem cell markers shows significant elevation (Table 1). The expression of Gli1 whose expressing cells are located to the entire suture mesenchyme and other osteogenic regions within the suture and play a role in suture morphogenesis^6^, is significantly different between the two cell populations (Table 1). As a control, Axin2 expression was much higher in the group of Axin2^+^/GFP^hi^ than Axin2^−^/GFP^−^ (Table 1). The results suggest that markers for bone marrow-derived skeletal stem cells may not be used for identification of SuSCs. As Axin2 is a well-known transcription target of β-catenin, it is possible that Wnt signalling is active in the Axin2^+^/GFP^hi^ population. We thus examined the potential Wnt ligand(s) required for maintenance of SuSCs. RT-PCR analysis showed that Wnt3a, 4, 7a, 10a, 10b and 11 are expressed in the suture mesenchyme (Supplementary Fig. 8a). We then performed gene-expression profile analysis of the Axin2^+^/GFP^hi^ and Axin2^−^/GFP^−^ cell populations by microarray. Wnt3a, 7a and 11 are significantly elevated in the Axin2-expressing cells (Supplementary Fig. 8b), implying their possible associations with the Axin2 activation in SuSCs.

### Stem cell characteristics of SuSCs

**Figure 6:**
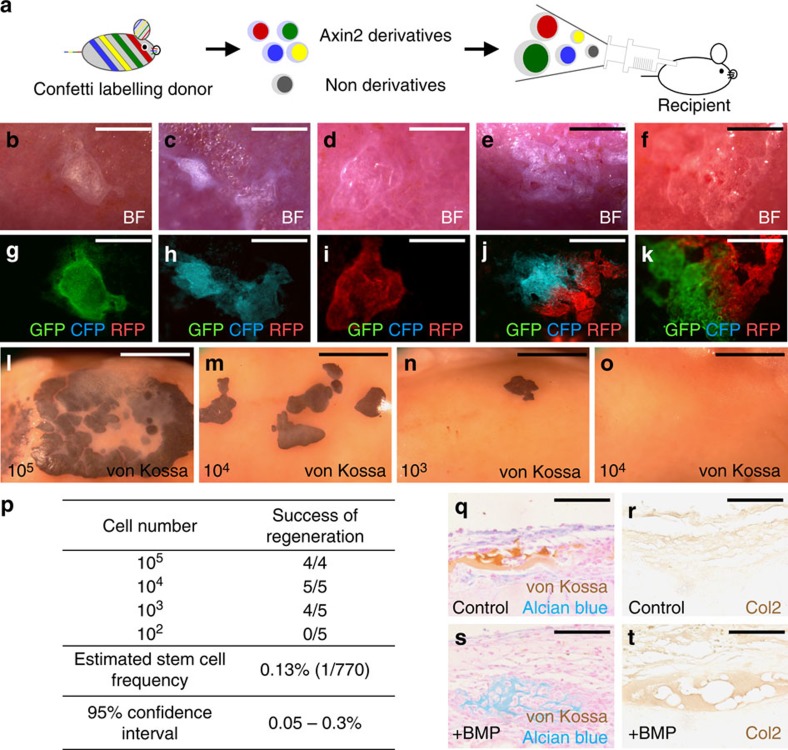
Bone regeneration examines the clonal expansion, stem cell frequency and multipotency of SuSCs *in vivo*. (**a**) Diagrams illustrate our strategies to examine stem cell characteristics of the Axin2-expressing cells at a single-cell level using *Axin2*^*Cre-Dox*^*; R26RConfetti*. Ectopic bone formation is assessed by gross evaluation (**b**–**f**), fluorescent imaging (**g**–**k**), von Kossa staining (**l**–**o**) in whole mounts. Limiting dilution analysis shows the success of bone regeneration with transplantation of 10^5^, 10^4^ and 10^3^, but not 10^2^ cells (**l**–**o**), providing a quantitative assessment for the stem cell frequency using ELDA software (**p**). The transplanted suture cells with (**s**–**t**) or without (**q**–**r**) addition of BMP2 were analyzed by double labelling of von Kossa and alcian blue (**q**,**s**) and immunostaining of type II collagen (**r**,**t**). Images are representatives of three (**b**–**k**,**q**–**t**), four (**l**) and five (**m**–**o**) independent experiments. Scale bar, 400 μm (**b**–**d**,**g**–**i**). Scale bar, 500 μm (**e**–**f**,**j**–**k**). Scale bar, 2 mm (**l**–**o**). Scale bar, 50 μm (**q**–**t**).

The abilities of a single Axin2-expressing cell to grow a colony and ectopic bone are essential features for the definition of stem cells. As the colony-forming ability was generally used for the study of bone marrow-derived skeletal stem cells^9^, we carried out a similar *in vitro* assay with cells isolated from the P28 *Axin2*^*Cre-Dox*^*; R26RlacZ* suture mesenchyme. Approximately 70% of colonies were derived from the Axin2-expressing cells, indicating their ability to form colonies from a single cell (Supplementary Fig. 9). However, using cells isolated from cortical bones of the femur and tibia, <2% of colonies are derivatives of the Axin2-expressing cells (Supplementary Fig. 4e,g). We were unable to detect any lacZ-positive colony when cells isolated from the bone marrow were used (Supplementary Fig. 4f,g). The per cent of colonies formed from the Axin2-expressing cells in the colony forming assay (Supplementary Fig. 9d; 69±3.2%, *n*=3, mean±s.e.m.) is lower than that of osteocytes formed from the Axin2-expressing cells in the transplantation analysis (Fig. 5f; 88±5.4%, *n*=3, mean±s.e.m.). This is likely owing to a higher contribution of skeletal precursors, which are progenitors but not stem cells, to form colonies *in vitro*^35^. Therefore, to perform a more rigorous test on the clonal expanding feature, we developed an *in vivo* assay using *R26RConfetti* mice. Cells, isolated from the P28 *Axin2*^*Cre-Dox*^*; R26RConfetti* suture mesenchyme, were implanted in the kidney capsule (Fig. 6a, Supplementary Fig. 1b). In this model, the Axin2-expressing cells were genetically labelled with random selection of GFP, YFP, RFP or CFP expression in a single cell, permitting its distinguishable identity and tracing. Three weeks after the implantation, we analyzed the regenerated bones, and found that they are labelled by a single fluorescent colour (Fig. 6b–d,g–i, Supplementary Fig. 10). Alternatively, the fluorescently labelled bones showed a patched pattern with clear boundaries between different colours (Fig. 6e,f,j,k, Supplementary Fig. 10). Examining the Axin2-expressing cells at a single cell level, not only provided compelling evidence for their clonal expansions *in vivo* but also indicated that they are *bona fide* stem cells in the suture mesenchyme. These SuSCs are pivotal for calvarial bone development, homoeostasis and injury repair. Furthermore, the bone regeneration study in the kidney capsule assayed the SuSC population which can be used for a faithful assessment of the stem cell number.

To determine the stem cell frequency, limiting dilution analysis was carried out. Various numbers of the suture mesenchymal cells were transplanted into the kidney capsule, followed by determination of the success rate in bone generation. Ectopic bones were consistently detected with transplantation of >10^4^ mesenchymal cells while the use of 10^2^ was never successful (Fig. 6l–o). The frequency of SuSCs was estimated at 0.13% using ELDA software (Fig. 6p). Furthermore, key characteristics of skeletal stem cells include their developmental ability to give rise to chondrocytes, found in the body skeleton. Although calvarial bones are formed via intramembranous ossification without formation of a cartilage intermediate, previous findings demonstrated that ectopic chondrogenesis can occur in the suture mesenchyme, leading to craniosynostosis mediated by endochondral ossification^5^. Activation of the canonical BMP pathway caused by disruption of Wnt and FGF signalling had been implicated in this mesenchymal cell fate switch^5^. To test the ability of SuSCs to differentiate into chondrocytes, we decided to manipulate their fate by stimulation of BMP signalling. The suture mesenchymal cells were implanted in the kidney capsule with or without the presence of BMP2. The transplanted SuSCs were able to generate bone but through different ossification processes. Seven days after the transplantation, double staining of von Kossa and alcian blue revealed mineral with no sign of cartilage in the controls (Fig. 6q). In contrast, cartilage but not mineral was detected in those with exogenous BMP2 (Fig. 6s). Through induction of BMP2, the Axin2-expressing SuSCs were able to alter their commitment from an osteogenic to a chondrogenic lineage (Fig. 6q–t). The *in vivo* evidence thus demonstrated that the identified SuSCs are capable of giving rise to both osteogenic and chondrogenic cell types.

### SuSCs facilitate bone healing through direct engraftment

**Figure 7:**
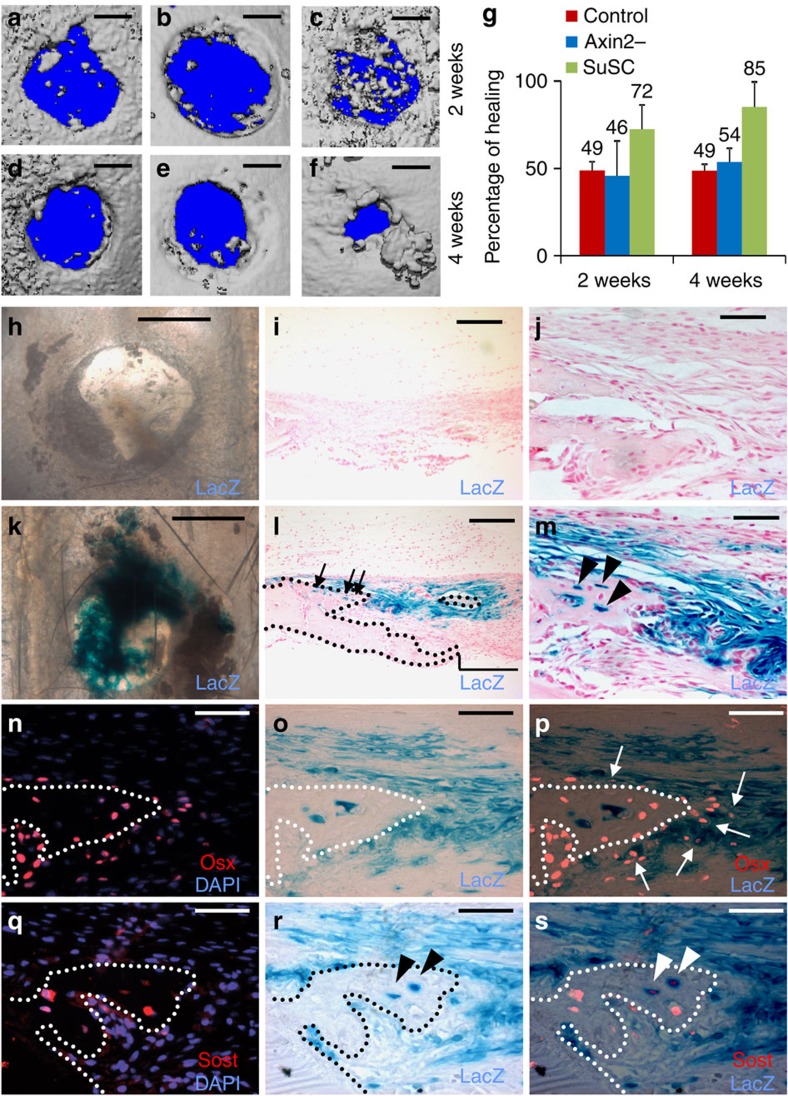
SuSCs improve bone healing through direct engraftment. The reparative ability of SuSC is examined by implanting without (**a**,**d**,**h**–**j**), or with suture mesenchymal cells containing Axin2^–^ (**b**,**e**) or Axin2^+^ (**c**,**f**,**k**–**s**) in the injury repair model. Two (**a**–**c**,**h**–**s**) and 4 (**d**–**f**) weeks after operation the healing process is assessed by micro CT (**a**–**f**), β-gal staining (**h**–**m**,**o**–**p**,**r**–**s**) and immunostaining of Osx (**n**,**p**) and Sost (**q**,**s**) in whole mounts (**a**–**f**,**h**,**k**) and sections (**i**–**j**,**l**–**s**). The graph shows the average per cent of healing at 2 and 4 weeks post operation (**g**, *n*=3, mean±s.e.m.; *P* value<0.05, Student *t*-test). Arrows, arrowheads and bracket indicate osteoprogenitors, osteocytes and unhealed area, respectively. Images are representatives of three independent experiments. Scale bar, 500 μm (**a**–**f**). Scale bar, 1 mm (**h**,**k**). Scale bar, 200 μm (**i**,**l**). Scale bar, 50 μm (**j**,**m**–**s**).

The identification of SuSCs prompted us to examine their potential application in stem cell-based therapy. As mesenchymal stem cells have been widely examined for their therapeutic potentials, it is important to explore this aspect of SuSCs. In addition, only short-term functional improvements were reported after the transplantation of mesenchymal stem cells most likely owing to a cell survival issue and/or none-cell autonomous effects^36^,^37^. With these critical obstacles in mind, we performed a series of experiments testing the ability of these newly identified SuSCs in tissue repair and regeneration using the injury repair model (Fig. 7). To examine their reparative ability, SuSCs, isolated from the P28 *Axin2*^*Cre-Dox*^*; R26RlacZ* suture mesenchyme with 3-day administration of doxycycline, were implanted into the injury site. We detected enhancements of the healing process at 2 and 4 weeks post operation (Fig. 7a–f). Transplantation of Axin2^−^ cells did not show significant improvement serving as control. When the repaired and unrepaired areas were measured, we found that the average per cent of healing improves significantly at 2 and 4 weeks post operation (Fig. 7g, *n*=3). We next examined if direct engraftment of the implanted cells into the regenerated bone occurs by β-gal staining analysis. SuSCs were able to engraft into the repaired bone (Fig. 7h–m) and develop into osteogenic cell types (Fig. 7n–s), suggesting that the direct engraftment ability of SuSCs provides benefits for skeletal repair.

## Discussion

This study provides compelling evidence that Axin2-expressing cells are a stem cell population capable of long-term self-renewal and differentiation into mature skeletogenic cell types *in vivo* during skeletal development and homeostastic maintenance. We have previously shown that the deletion of Axin2 causes premature suture closure and craniosynostosis in mice^27^. Furthermore, Axin2 is developmentally regulated in the posterior frontal suture^5^, which is the only suture undergoing normal fusion processes and lost mesenchymal tissues. The Axin2-expressing cells are reduced in coordination with frontal suture closure. Therefore, the current and previous studies clearly demonstrate the importance of Axin2 and the Axin2-expressing cell population in suture development and maintenance^5^,^27^. The Axin2-expressing cells are also highly responsive to calvarial injury. Genetic cell labelling analysis shows that 1 month after tracing, 46% of cells residing in the skeletogenic mesenchyme are their derivatives, with increases to 98% during damage-induced repair. This finding suggests that the Axin2-expressing cell population is the main cell type in the skeletogenic mesenchyme responding to the injury. To date, the only other marker able to mark the cell population contributing to calvarial maintenance and injury repair is Gli1 (ref. 6). Gene-profiling analysis shows high levels of Gli1 expression in the Axin2-expressing cells, suggesting an overlap of these two cell populations. However, Gli1 appears to mark the entire suture mesenchyme with exception of a few cells at the osteogenic fronts in adults. In contrast, Axin2 only expresses in about 15% of the mesenchymal cells, is highly restricted to the midline of the suture, and is co-localized with the slow cycling cells/LRCs, providing a more-specific approach to isolate the SuSC population. On the basis of Axin2 expression that coincides with the LRCs restricted to the suture midline in the early neonatal period (after postnatal day 9), we successfully identify SuSCs, contributing to calvarial development and homeostastic maintenance. This feature is especially important for elucidating pathogenic mechanisms of craniosynostosis and other craniofacial disorders associated with aberrant calvarial morphogenesis.

Using Axin2 as a marker, we are able to isolate a major SuSC population for the first time. A combination of cell tracing and kidney capsule transplantation demonstrates that this cell population contains SuSCs promoting calvarial bone formation. *In vivo* clonal analysis of the Axin2-expressing cells at the single-cell level further demonstrates that they undergo clonal expansion as a patch of cells sharing a common origin to generate the ectopic bone. If the Axin2-expressing cells are osteoprogenitors but not stem cells, we would detect ectopic bones developed from a mixed population of cells with distinct origins. However, no mixture of cells derived from different clones has been detected, suggesting that bone regeneration ability in the renal capsule mainly comes from the stem cell properties. Furthermore, these stem cells can become chondrocytes although osteoblast cell types are their normal developmental fate. This multipotency provides additional proof of their stem cell identity. The demonstration of SuSC characteristics preserved in the transplantation thus permits an estimation of the stem cell number using limiting dilution analysis. Although ∼15% of suture mesenchymal cells express Axin2, only 0.13% of them are SuSCs according to the ectopic bone formation with limiting dilution analysis. About 1% of the Axin2-expressing cells are SuSCs. Therefore, the use of kidney capsule transplantation with limiting dilution analysis provides a functional and much more precise method to determine the stem cell frequency. This powerful assay can not only be used to rigorously test true skeletal stem cells, but also potentially uncover skeletal diseases caused by stem cell abnormalities.

Although Axin2 is an excellent marker for identification of SuSCs, the Axin2-expressing cell pool appears to have limited contributions to maintenance of the body skeleton. Clonal expansion analysis also shows that the Axin2-expressing cells isolated from the cortical bone or bone marrow give rise to <2% of the colonies. Transplantation of the cortical bone cells reveals limited, but definitively not predominant, contributions of the Axin2-expressing cells to ectopic bone formation in the renal capsule. Furthermore, a very low number of Axin2-expressing cells and their descendants can be detected in the long bone after 1-year tracing, implying that Axin2 is not a good marker for skeletal stem cells residing in the long bone. Consistent with these observations, previously identified bone marrow-derived skeletal stem cell markers, except for Lepr, are not significantly elevated in the Axin2-expressing cells isolated from the suture mesenchyme. Contrary to the previous notion^38^, our data do not support the use of Axin2 as a marker for the bone marrow-derived skeletal stem cells because the majority of them are not included in the Axin2-expressing cell population. Differential localizations of the Axin2+ and Gli1+ cells may also contribute to the regulatory mechanism underlying skull homoeostasis. Gli1+ cells provide calvarial maintenance near the centre of bone plates containing bone marrows^6^ where Axin2+ cells have relatively little contribution. It is possible that distinct stem cell populations are responsible for different calvarial areas. The Axin2+ cells are not associated with the marrow region, thus not included in the stem cell population for the body skeleton. Furthermore, transplanted SuSCs form intramembranous bones resembling the calvarial bone plates which are morphologically distinguishable from the endochondral bone generated by cells isolated from the long bone. Similar phenomena have also been described by transplantation of embryonic cells derived from the calvaria and long bones^34^. Our findings suggest a unique stem cell population, called SuSC, for calvarial bone formation mediated by intramembranous ossification.

As an emerging field, tissue engineering, combining an interdisciplinary study of stem cells, materials and growth factors, is actively investigated in preclinical and clinical trials^39^,^40^. For craniofacial bone reconstruction, bone grafts collected from the craniofacial skeleton have demonstrated superior volumetric maintenance and survival compared with those from the endochondral bones^15^. Current identification, isolation and characterization of the Axin2-expressing SuSCs have advanced our knowledge base of calvarial bone development and homoeostasis. Their future study promises new insights into pathogenic mechanisms of skeletal disease. The SuSCs could be an ideal cell type for cell-based craniofacial bone therapy as they possess abilities to engraft, differentiate into skeletogenic cell types, generate bones and enhance repair processes. Further analysis of SuSCs provides outstanding opportunities to improve craniofacial repair and reconstruction, leading to future advancement in regenerative medicine.

## Methods

### Animals and models

The *Axin2-rtTA*, *TRE-H2BGFP*, *TRE-Cre*, *R26RlacZ*, *R26RTomato*, *R26RConfetti*, *SCID* mouse strains and genotyping methods were reported previously^5^,^28^,^41^,^42^,^43^,^44^,^45^. To generate *Axin2*^*GFP*^ mouse strain^5^,^46^, mice carrying the *Axin2-rtTA* and *TRE-H2BGFP* transgenes were obtained and treated with doxycycline (2 mg ml^−1^ plus 50 mg ml^−1^ sucrose) for 3 days as described^28^,^41^,^42^. The *Axin2*^*Cre-Dox*^ mouse strain was created by obtaining mice carrying *Axin2-rtTA* and *TRE-Cre* transgenes. The expression of Cre in the Axin2-expressing cells was then induced by doxycycline treatment^5^,^29^. Both male and female mice were used in this study. Care and use of experimental animals described in this work comply with guidelines and policies of the University Committee on Animal Resources at the University of Rochester.

For kidney capsule transplantation, cells were pelleted at 200 g and re-suspended in 10 μl Matrigel (BD Biosciences, San Jose, CA) with or without BMP2 (100 μg ml^−1^), followed by injection into 8–12–week-old *SCID* mice. The calvarial injury model was established by creation of a circular lesion about 1.4 mm in diameter using a micro-drill. To administer cells into the injury site, an implant of 5 μl Matrigel containing donor cells was placed on top of the circular lesion immediately after the injury.

### Cells and genes

Primary suture mesenchymal cells containing SuSCs were isolated from the calvaria^5^,^27^. Specifically, an ∼1.5-mm wide tissue containing sagittal suture and its adjacent parietal bones were collected. Next, the parietal bones were separated from the suture to expose the suture mesenchyme, which is subsequently incubated with 0.2% collagenase in PBS at 37 °C for 1.5 h. The dissociated cells were then filtered through a 40 μm strainer, followed by re-suspension in DMEM media for transplantation analysis, or in DMEM containing 5% FBS for cell sorting. To purify Axin2^+^/GFP^hi^ and Axin2^−^/GFP^−^ populations, suture mesenchymal cells isolated from *Axin2*^*GFP*^ mice were subjected to cell sorting according to the intensity of GFP using FACSAria-II (BD Biosciences). To isolate the osteoprogenitors from the long bone, tibia and femur were dissected by careful removal of the skins and muscles. After epiphyses were removed, the remaining bones were crushed with a mortar to extract marrows which were then washed with PBS and incubated with 0.1% collagenase in PBS for 1 h at 37 °C. The dissociated cells were subsequently collected using a 40 μm strainer for kidney capsule transplantation analysis. For colony-forming assay, 10^3^–10^4^ cells isolated from the suture mesenchyme were cultured in the 10 cm plate with DMEM media containing 20% FBS for 2 weeks. To examine gene expression, total RNA isolated from the suture mesenchyme was subject to the first-strand cDNA synthesis (iScript, Bio-Rad, Hercules, CA), followed by semi-quantitative or real-time (CFX96 system, Bio-Rad) PCR amplification (40 cycles, 95 °C for 15 s, 61 °C, 30 s, 72°C for 30 s). For real-time PCR, the method by Livak and Schmittgen^47^ was applied to calculate the relative RNA expression using GAPDH as an internal calibrator. The primer sets specific for the target sequences are listed in Supplementary Table 1.

### Staining and analysis

Skull preparation, fixation and embedding for paraffin and frozen sections were performed^5^,^27^,^29^,^48^. Samples were subjected to hematoxylin/eosin staining for histology, Pentachrome staining, GFP analysis, β-gal staining, van Kossa staining or immunological staining with avidin:biotinylated enzyme complex^5^,^27^,^28^,^41^,^48^,^49^,^50^,^51^,^52^. For whole-mount von Kossa staining, the transplanted kidneys were fixed with 2% paraformaldehyde and 0.02% NP-40 at room temperature for 1 h, followed by incubation with 1% silver nitrate under ultraviolet light for 30 min, and then 5% sodium thiosulfate for 5 min. The immunological staining was visualized by enzymatic colour reaction or fluorescence according to the manufacture's specification (Vector Laboratories, Burlingame, CA). Double labelling analysis was performed by immunological staining on the β-gal-stained sections^48^. Rabbit polyclonal antibodies Osterix (ab22552, Abcam, Cambridge, MA; 1:200); goat polyclonal antibodies SOST (AF1589, R&D Systems, Minneapolis, MN; 1:20); mouse monoclonal antibodies Collagen II (ms-235, Neomarkers, Fremont, CA; 1:50) were used in these analyses. Images were taken using Zeiss Axio Observer microscope (Carl Zeiss, Thornwood, NY).

### Statistics and reproducibility

R software version 3.2.1 or Microsoft Excel 2010 was used for statistical analysis. The significance was determined by two-sided Student's *t*-tests. A *P*-value <0.05 were considered statistically significant. Before performing the *t*-tests, normality of the data distribution was first validated by Shapiro–Wilk normality test. Analysis of samples by μCT was performed by a technician who is blinded to the condition. No randomization, statistical method to predetermining the sample size and inclusion/exclusion criteria defining criteria for samples were used. At least three independent experiments were performed for statistical analyses of the animal tissues described in figure legends. Statistical data were presented as mean±s.e.m. The stem cell frequency was examined by Extreme Limiting Dilution Analysis (ELDA) software ( http://bioinf.wehi.edu.au/software/elda/) with validation of the likelihood ratio test for a single-hit model^53^.

## Additional information

**Accession codes:** Microarray data have been deposited at the NCBI, the GEO database under the accession code GSE74849.

**How to cite this article:** Maruyama, T. *et al.* Stem cells of the suture mesenchyme in craniofacial bone development, repair and regeneration. *Nat. Commun.* 7:10526 doi: 10.1038/ncomms10526 (2016).

## Supplementary Material

Supplementary Information Supplementary Figures 1-10 and Supplementary Table 1.

## Figures and Tables

**Table 1:** The expression of Lepr and Gli1 is significantly elevated in SuSCs.

**Gene**	**Microarray**		**qPCR**	
	**Fold change**	***P***-**value (*****n*****=3)**	**Fold change**	***P***-**value (*****n*****=6)**
**Lepr**	**1.32**	**0.0112**	**18.57**	**0.0202**
**Gli1**	**1.27**	**0.0252**	**1.37**	**0.0132**
Nestin	1.30	0.0677	0.67	<0.0001
Gremlin1	1.07	0.4472	UD	UD
Mcam/CD146	1.04	0.8593	1.02	0.5294
**Axin2**	**1.50**	**0.0055**	**2.21**	**0.0028**

SuSCs, suture stem cell; UD, undetectable.

Differential expression of the skeletal stem cell markers in the Axin2-expressing cell/SuSC population with high levels of GFP (Axin2^+^/GFP^hi^) and the non-expressing cell population negative for GFP (Axin2^−^/GFP^−^). Statistics were performed by two-sided Student's *t*-tests.

Bold values indicate statistical significance for the changes.
