# Supplementary material for: Stem cells of the suture mesenchyme in craniofacial bone development, repair and regeneration
Source: Nat Commun. 2016 Feb 1;7:10526. doi: 10.1038/ncomms10526 (PMC4740445; doi:10.1038/ncomms10526)
Supplement: Supplementary Information — Supplementary Figures 1-10 and Supplementary Table 1. [file ncomms10526-s1.pdf]

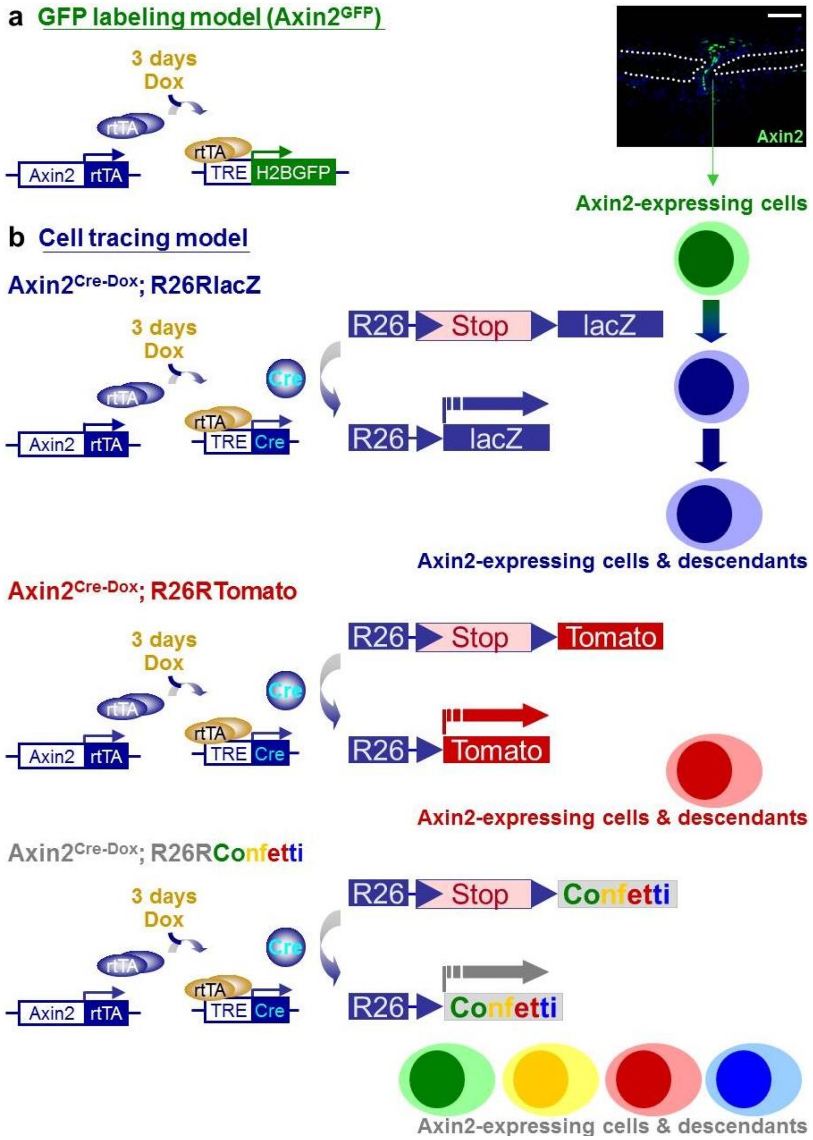

Supplementary Figure 1. Mouse genetic models used for identification of Axin2-expressing cells and their derivatives. Schematic representations illustrate genetic cell labeling strategies to identify the Axin2-expressing cells by GFP analysis (a, GFP labeling model), and the Axin2-expressing cells and their derivatives by β-gal staining or fluorescent imaging (b, Cell tracing model). GFP analysis of the *Axin2-rtTA; TRE-H2BGFP* mice at P9 shows the Axin2-expressing cells in the midline of the suture mesenchyme (a, n=3) whose fate can be further traced in the cell tracing model (b). Scale bar, 100 μm (a).

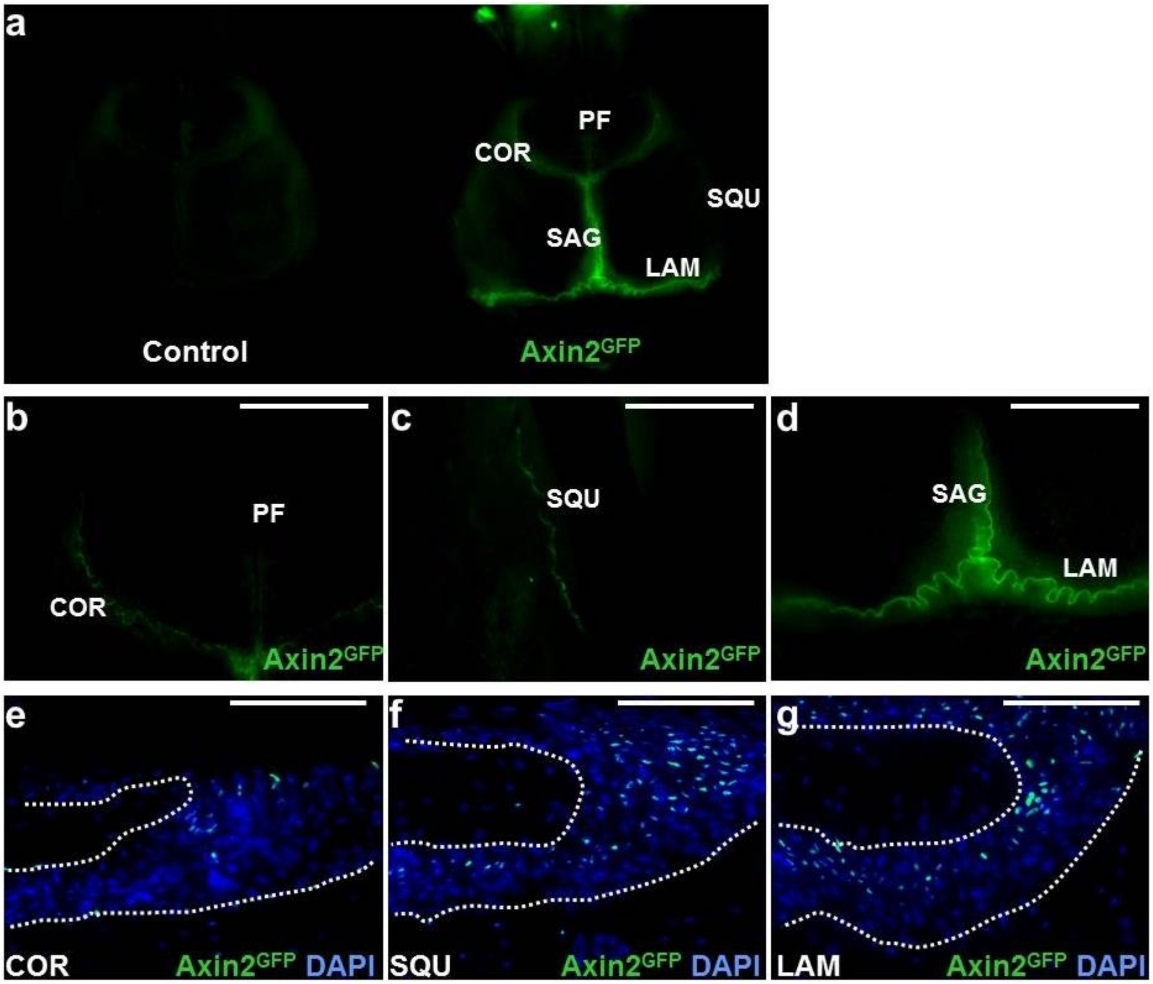

Supplementary Figure 2. Axin2-expressing cells in calvarial sutures. The presence of Axin2-expressing cells is identified using *Axin2*^{GFP} in whole mounts and sections. Sutures: COR, coronal; LAM, lambdoidal; PF, posterior frontal; SAG, sagittal; SQU, squamosal. Images show representative data from three independent experiments. Scale bars, 2 mm (b-d); 100 µm (e-g).

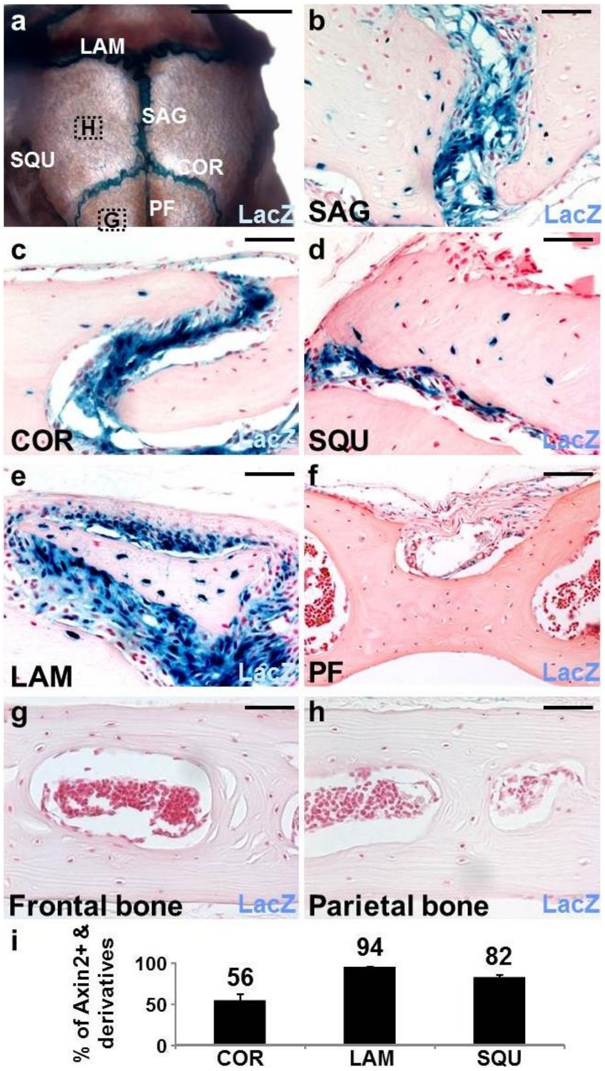

Supplementary Figure 3. Axin2-expressing cells in homeostastic maintenance of the calvarial bones. (a) Using *Axin2$^{Cre-Dox}$; R26RlacZ* mice, the Axin2-expressing cells and their descendants were identified in the coronal (COR), lambdoidal (LAM) and squamosal (SQU) sutures in addition to the sagittal suture by β-gal staining in whole mount (a) and sections (b-h). No lacZ positive cells were found in posterior frontal suture (PF), and the central region of frontal and parietal bones further away from the suture mesenchyme (f-h). Enlargements of the insets in panel a are shown in g and h Statistical analysis indicates the ratio of the lacZ positive cells after more than 1 year of tracing (I, ~250 cells counted, n=3, mean $\pm$ SEM). Scale bars, 4 mm (a); 50 µm (b-h).

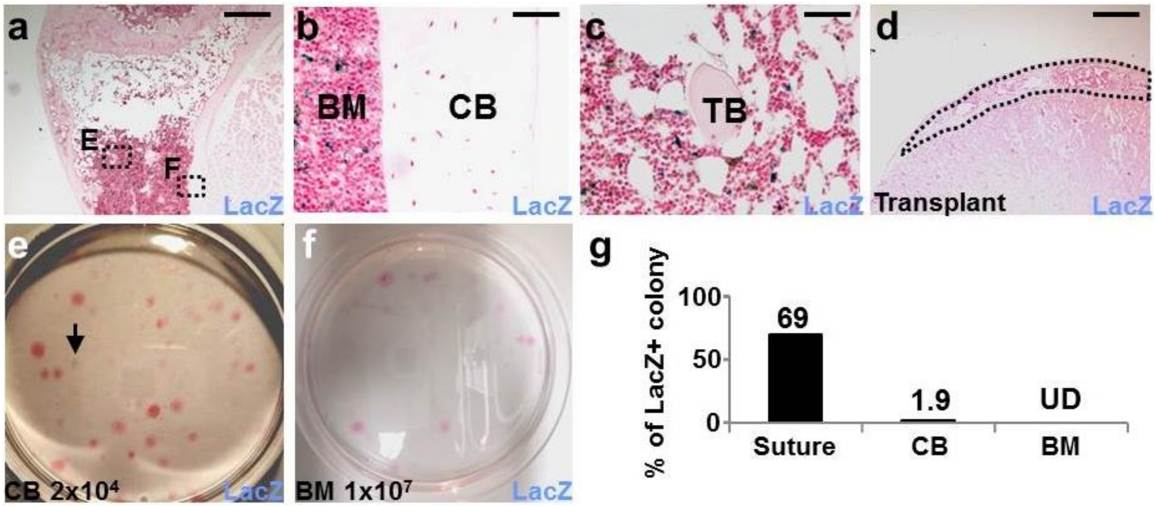

Supplementary Figure 4. Axin2 is not a marker for skeletal stem cells in the body skeleton. The Axin2-expressing cells and their descendants were analyzed in the *Axin2^Cre-Dox^; R26RlacZ* femur after more than 1 year tracing (a-c). Osteocytes appeared to be negative for β-gal staining while few positive cells were only found in the bone morrow. Cell tracing analysis was also performed for cells isolated from the P28 cortical bone (d-e) and bone marrow (f) in kidney capsule transplantation (d) and colony forming (e-f) assays. Broken lines highlight the ectopic bone. Statistical analysis indicates the ratio of the lacZ positive colony (g). BM, bone marrow; CB, cortical bone; TB, trabecular bone; UD, undetectable. Images are representatives of three independent experiments. Scale bars, 500 μm (a, d); 50 μm (b-c).

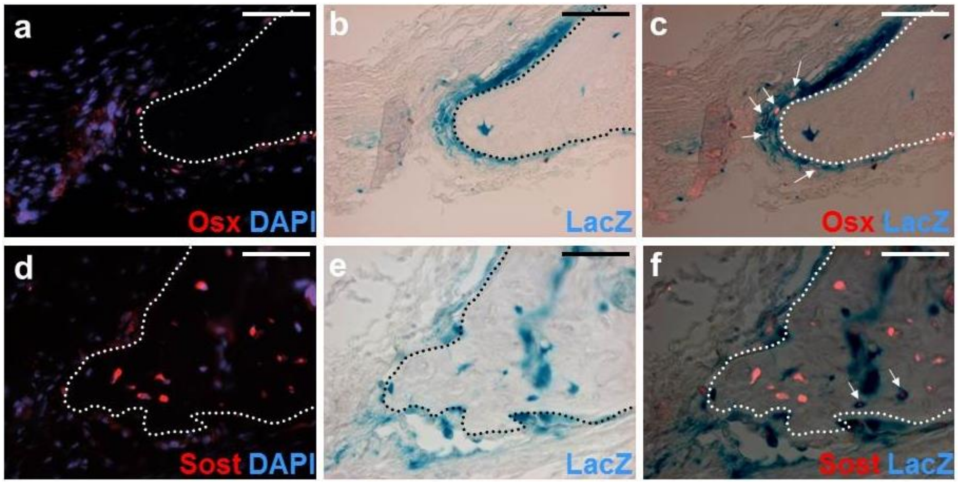

Supplementary Figure 5. Axin2-expressing cells contribute to skeletal injury repair though direct differentiation into osteoblasts and osteocytes. Immunostaining analysis of Osx (a) and Sost (d) identifies osteogenic cell types after 4 week tracing. The Axin2-expressing cells and their derivatives were identified by β-gal staining (b, e). Double labeling analysis reveals the expression of osteogenic markers in the Axin2-expressing cells and their derivatives (c, f). Arrows indicate the lacZ positive cells expressing the osteogenic marker. Genotypes: *Axin2rtTA; TRE-Cre; R26R*. Images are representatives of three independent experiments. Scale bars, 50 μm (a-f).

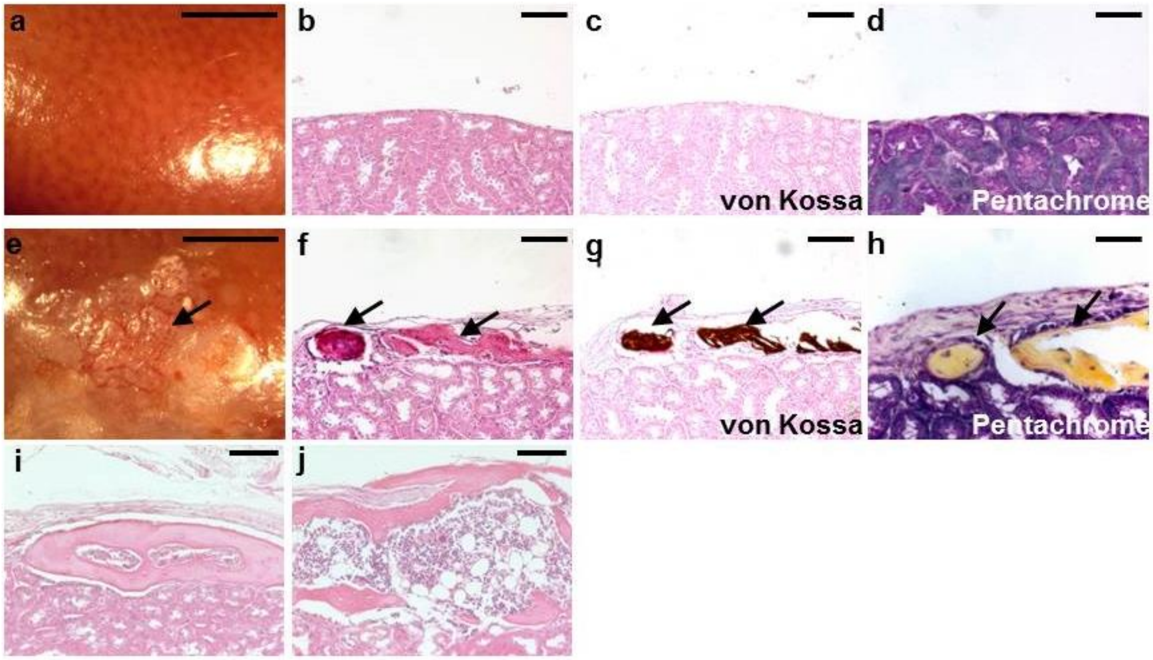

Supplementary Figure 6. Bone regenerating ability is determined by transplantation at the ectopic site. Kidney capsule transplantations with (e-h) or without (a-d) inclusion of 10^5 cells isolated from the suture mesenchyme (e-i), or the femur and tibia containing cortical bone cells (j) demonstrate their ability to form bones. Ectopic bone formation is assessed by gross evaluation (a, e), hematoxylin and eosin staining (b, f, i-j), von Kossa staining (c, g) and pentachrome staining (d, h) in whole mounts (a, e) and sections (b-d, f-j). Arrows indicate the regenerated bone in kidney capsule. Images are representatives of three independent experiments. Scale bars, 4 mm (a, e); 100 µm (b-c, f-g, i-j); 50 µm (d, h).

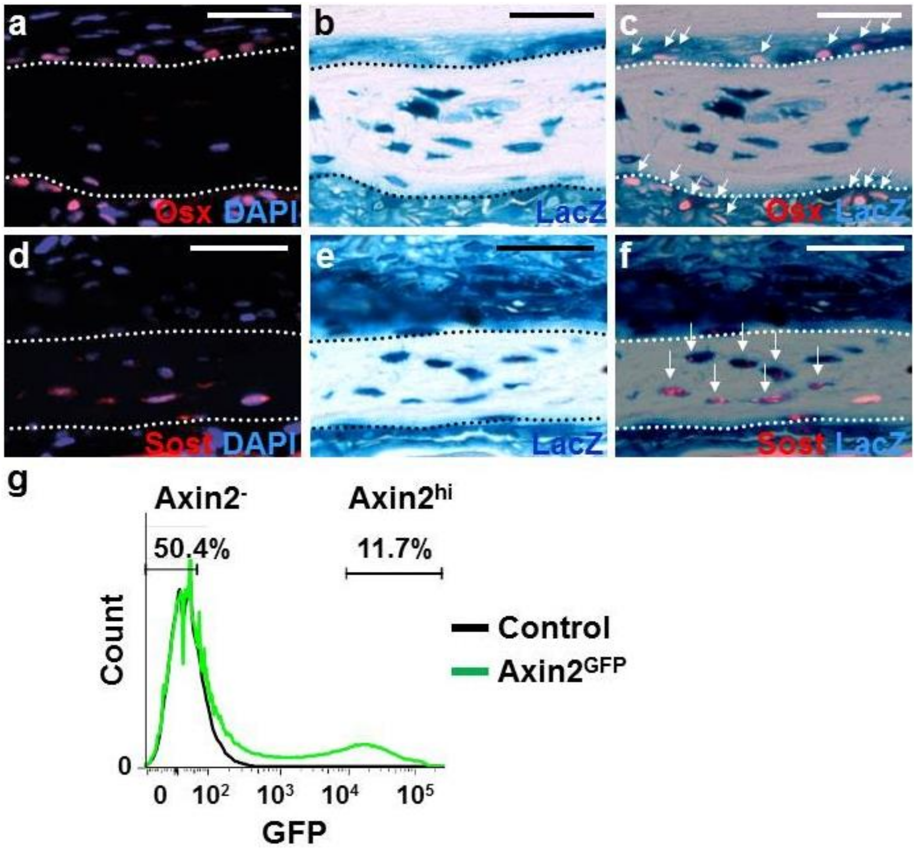

Supplementary Figure 7. Axin2-expressing cells contain SuSCs capable of producing osteoblasts and osteocytes in bone regeneration analysis. Ectopic bone formation was detected in the kidney capsule two weeks after transplantation with cells isolated from the suture mesenchyme of *Axin2*$^{Cre\text{-}Dox}$*; R26RlacZ* at P28. Sections of the regenerated bone were stained with osteogenic markers (a, Osx; d, Sost), as well as β-gal (b, e) to reveal the cellular identity as well as their origination from the Axin2-expressing cells, respectively. Double labeling analysis shows the expression of osteogenic markers in the Axin2-expressing cells and their derivatives (c, f). Arrows indicate the cells double positive for the osteogenic marker and lacZ. (g) Graph indicates the GFP-based sorting of Axin2^{+}/GFP^{hi} and Axin2^{-}/GFP^{-} used in the regeneration analysis performed in Figure 5G-I. Images are representatives of three independent experiments. Scale bars, 50 μm (a-f).

a

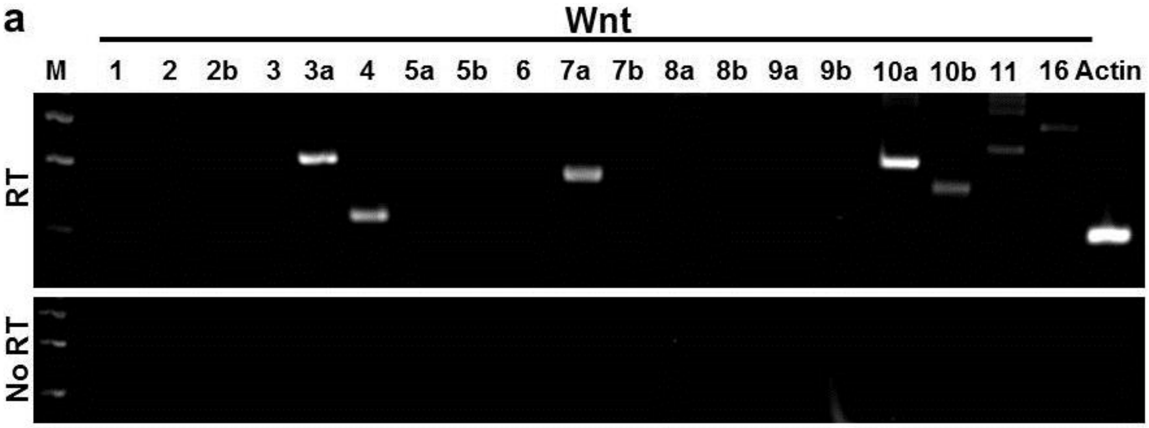

b

Gene	Axin2+ vs. Axin2- cells	
	Fold change	*p* value (n=3)
**Wnt3a**	**1.66**	**0.022**
Wnt4	1.10	0.444
**Wnt7a**	**1.90**	**0.014**
Wnt10a	1.19	0.386
Wnt10b	1.11	0.303
**Wnt11**	**1.63**	**0.022**

Supplementary Figure 8. Expression of Wnt ligands is enhanced in SuSCs. (a) RT-PCR analysis detects the transcripts of Wnt3a, 4, 7a, 10a, 10b and 11 but not Wnt1, 2, 2b, 3, 5a, 5b, 6, 7b, 8a, 8b, 9a, 9b and 16 in the suture mesenchyme. (b) Differential expression of Wnt ligands in the Axin2-expressing cell/SuSC population with high levels of GFP ($Axin2^+/GFP^{hi}$) and the non-expressing cell population negative for GFP ($Axin2^-/GFP^-$). Statistical analyses were performed by two-sided student's t-tests.

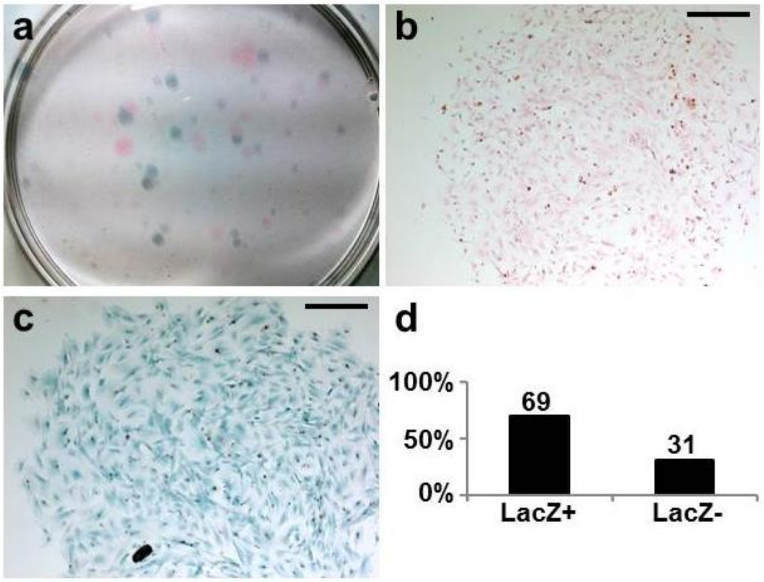

Supplementary Figure 9. Clonogenic ability of Axin2-expressing cells *in vitro*. (a) Cells isolated from the P28 Axin2$^{Cre-Dox}$; R26RlacZ suture were cultured for two weeks followed by β-gal staining. Higher magnification showed cells within a colony derived from non Axin2-expressing (b) and Axin2-expressing (c) cells. The ratio of the lacZ positive (+) and negative (-) colonies was indicated (d). Images are representatives of three independent experiments. Scale bars, 500 μm (b-c).

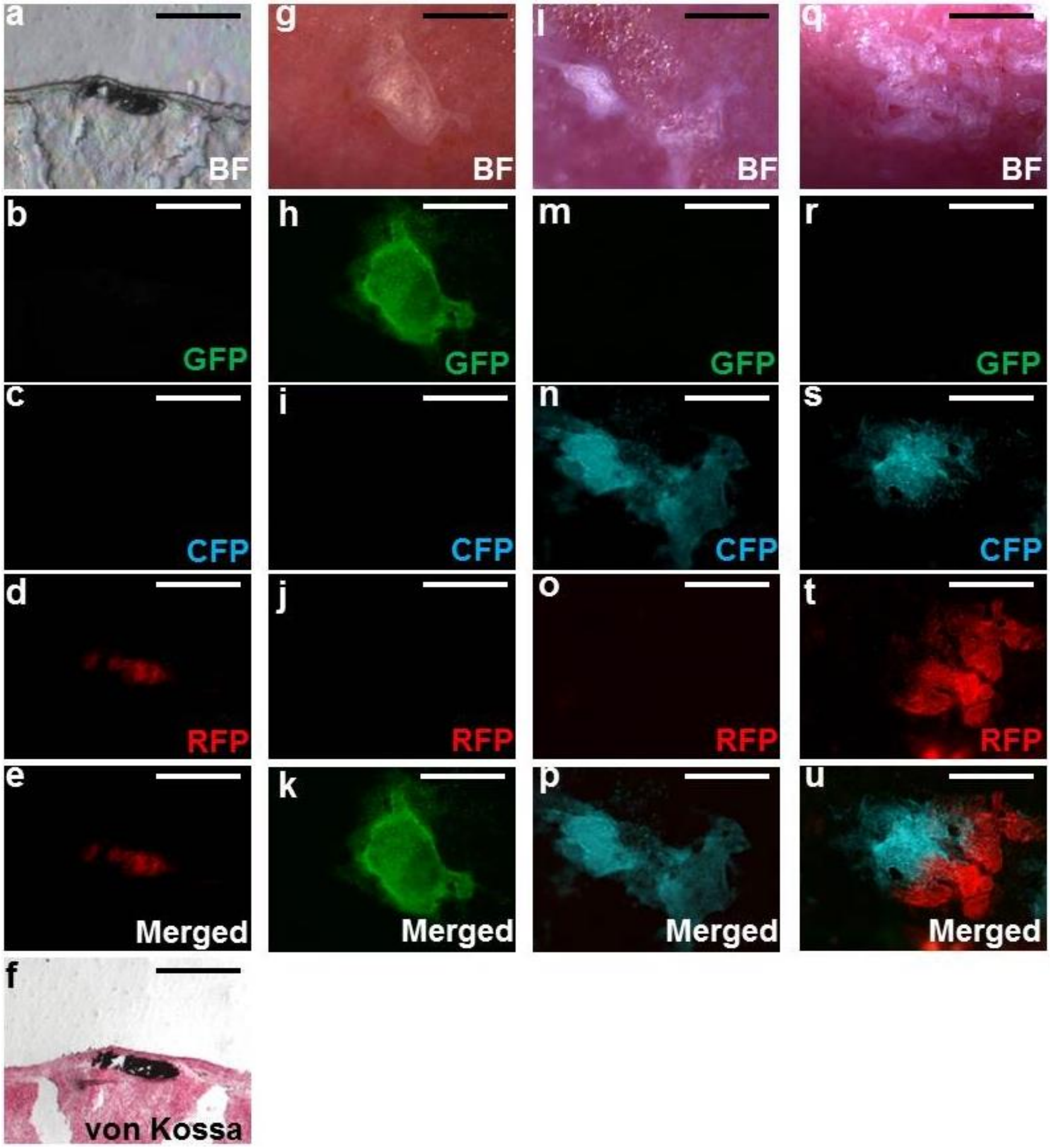

Supplementary Figure 10. Clonal expansion analysis of Axin2-expressing cells *in vivo*. Transplantation of cells isolated from the *Axin2*$^{Cre\text{-}Dox}$*; R26RConfetti* into the kidney capsule analyzes their expansion and developmental potentials. The regenerated bones were examined by bright field (BF), fluorescent imaging with the GFP, CFP or RFP filter, merged GFP/CFP/RFP images (Merged) and von Kossa staining as indicated in whole mounts (g-u) and sections (a-f). Images are representatives of three independent experiments. Scale bars, 400 µm (a-p); 500 µm (q-u).

	**Forward primer**	**Reverse primer**
Lepr	CATTTCCGCTTCAATATCAGG	CCAGCAGAGATGTAGCTGAGAC
Nestin	CTGCAGGCCACTGAAAAGTT	TCTGACTCTGTAGACCCTGCTTC
Mcam	GGAAACTACCATCACACTGTCAAGT	TGGATTCACTTGAACATCAGACA
Gremlin1	GACCCACGGAAGTGACAGA	CCCTCAGCTGTTGGCAGTAG
Gli1	AGGAATTCGTGTGCCATTG	TCCGACAGCCTTCAAACG
Gapdh	AGCTTGTCATCAACGGGAAG	TTTGATGTTAGTGGGGTCTCG
β-actin	AAGGCCAACCGTGAAAAGAT	GTGGTACGACCAGAGGCATAC
Wnt1	GGTTTCTACTACGTTGCTACTGG	GGAATCCGTCAACAGGTTCGT
Wnt2	CTCGGTGGAATCTGGCTCTG	CACATTGTCACACATCACCCT
Wnt2b	CCGACGTGTCCCCATCTTC	GCCCCTATGTACCACCAGGA
Wnt3	AGGAGTGCCAGCATCAGTTC	ACTTCCAGCCTTCTCCAGGT
Wnt3a	CTGGCAGCTGTGAAGTGAAG	TGGGTGAGGCCTCGTAGTAG
Wnt4	AGACGTGCGAGAAACTCAAAG	GGAACTGGTATTGGCACTCCT
Wnt5a	CAACTGGCAGGACTTTCTCAA	CATCTCCGATGCCGGAACT
Wnt5b	CTGCTGACTGACGCCAACT	CCTGATACAACTGACACAGCTTT
Wnt6	GCAAGACTGGGGGTTCGAG	CCTGACAACCACACTGTAGGAG
Wnt7a	GGTGCGAGCATCATCTGTAA	TCCTTCCCGAAGACAGTACG
Wnt7b	TTTGGCGTCCTCTACGTGAAG	CCCCGATCACAATGATGGCA
Wnt8a	GGGAACGGTGGAATTGTCCTG	GCAGAGCGGATGGCATGAA
Wnt8b	CCCGTGTGCGTTCTTCTAGTC	AGTAGACCAGGTAAGCCTTTGG
Wnt9a	GGCCCAAGCACACTACAAG	AGAAGAGATGGCGTAGAGGAAA
Wnt9b	CTGGTGCTCACCTGAAGCAG	CCGTCTCCTTAAAGCCTCTCTG
Wnt10a	GCTCAACGCCAACACAGTG	CGAAAACCTCGGCTGAAGATG
Wnt10b	GCGGGTCTCCTGTTCTTGG	CCGGGAAGTTTAAGGCCCAG
Wnt11	GCTGGCACTGTCCAAGACTC	CTCCCGTGTACCTCTCTCCA
Wnt16	CAGGGCAACTGGATGTGGTT	CTAGGCAGCAGGTACGGTT

Supplementary Table 1. Primer sets used for RT-PCR analysis.
